# Biochip-Based Detection of *KRAS* Mutation in Non-Small Cell Lung Cancer

**DOI:** 10.3390/ijms12128530

**Published:** 2011-11-29

**Authors:** Gernot Kriegshäuser, Gerhild Fabjani, Barbara Ziegler, Sabine Zöchbauer-Müller, Adelheid End, Robert Zeillinger

**Affiliations:** 1 ViennaLab Diagnostics GmbH, 1120 Vienna, Austria; E-Mail: kriegshauser@viennalab.co.at; 2 Molecular Oncology Group, Department of Obstetrics and Gynecology, Medical University Vienna, 1090 Vienna, Austria; E-Mails: gerhild.fabjani@gmx.at (G.F.); barbara.ziegler@meduniwien.ac.at (B.Z.); 3 Ludwig Boltzmann Gesellschaft, Cluster Translational Oncology, 1090 Vienna, Austria; 4 Division of Oncology, Department of Internal Medicine I, Medical University of Vienna, 1090 Vienna, Austria; E-Mail: sabine.zoechbauer-mueller@meduniwien.ac.at; 5 Department of Cardiothoracic Surgery, Medical University of Vienna, 1090 Vienna, Austria; E-Mail: adelheid.end@meduniwien.ac.at

**Keywords:** non-small cell lung cancer, *KRAS*, mutation detection, biochip hybridization

## Abstract

This study is aimed at evaluating the potential of a biochip assay to sensitively detect *KRAS* mutation in DNA from non-small cell lung cancer (NSCLC) tissue samples. The assay covers 10 mutations in codons 12 and 13 of the *KRAS* gene, and is based on mutant-enriched PCR followed by reverse-hybridization of biotinylated amplification products to an array of sequence-specific probes immobilized on the tip of a rectangular plastic stick (biochip). Biochip hybridization identified 17 (21%) samples to carry a *KRAS* mutation of which 16 (33%) were adenocarcinomas and 1 (3%) was a squamous cell carcinoma. All mutations were confirmed by DNA sequencing. Using 10 ng of starting DNA, the biochip assay demonstrated a detection limit of 1% mutant sequence in a background of wild-type DNA. Our results suggest that the biochip assay is a sensitive alternative to protocols currently in use for *KRAS* mutation testing on limited quantity samples.

## 1. Introduction

Lung cancer is one of the most common human cancers and is the leading cause of cancer death worldwide, with non-small cell lung cancer (NSCLC) accounting for up to 85% of all cases [1]. In lung carcinoma, epidermal growth factor receptor (EGFR) is more abundantly expressed than in adjacent normal lung [2]. EGFR signaling pathways include downstream GTPases encoded by *RAS* genes, and the incidence of activating *KRAS* mutation in patients with NSCLC ranges from 8% to 24% with most mutations located in codons 12 and 13 at exon 2 [3,4]. Interestingly, *KRAS* mutations are frequently found in histologically normal tissues near tumors, suggesting that such mutations may represent an early event in lung carcinogenesis [5]. Somatic gain-of-function mutations in the tyrosine kinase domain of the *EGFR* have been identified in up to 40% of NSCLC patients [6], and these mutations are associated with sensitivity to small-molecule tyrosine kinase inhibitors like gefitinib or erlotinib [7]. *EGFR* and *KRAS* mutations have been reported to be mutually exclusive, and NSCLC patients carrying a *KRAS* mutation do not respond to tyrosine kinase inhibitors [8]. Additionally, *KRAS* mutation seem to be associated with unfavorable outcomes making *KRAS* both a predictive and a prognostic marker in NSCLC [3], although its predictive role is still inconclusive as indicated by several recent studies [4].

Recently, a low-density biochip assay, designed for the sensitive detection of 10 mutations in codons 12 and 13 of the *KRAS* gene (Val12, Asp12, Leu12, Ser12, Ala12, Ile12, Cys12, Arg12, Cys13, Asp13) has successfully been introduced to *KRAS* mutation screening in ovarian cancer [9–11]. The assay is based on peptide nucleic acid (PNA)-mediated mutant-enriched PCR and reverse-hybridization of amplification products to oligonucleotide probes immobilized on the tip of a rectangular plastic stick (biochip) [9]. The biochip assay demonstrated an analytical sensitivity of 0.1% using dilutions of genomic DNA prepared from tumor cell lines [9], whereas a loss of sensitivity was observed when the assay was performed on formalin-fixed paraffin-embedded (FFPE)-extracted DNA [11].

This study is aimed at evaluating the potential of the biochip assay to sensitively detect mutant *KRAS* in 81 NSCLC samples, and the presence of *KRAS* mutation was then verified by DNA sequencing.

## 2. Results and Discussion

**Figure 1:**
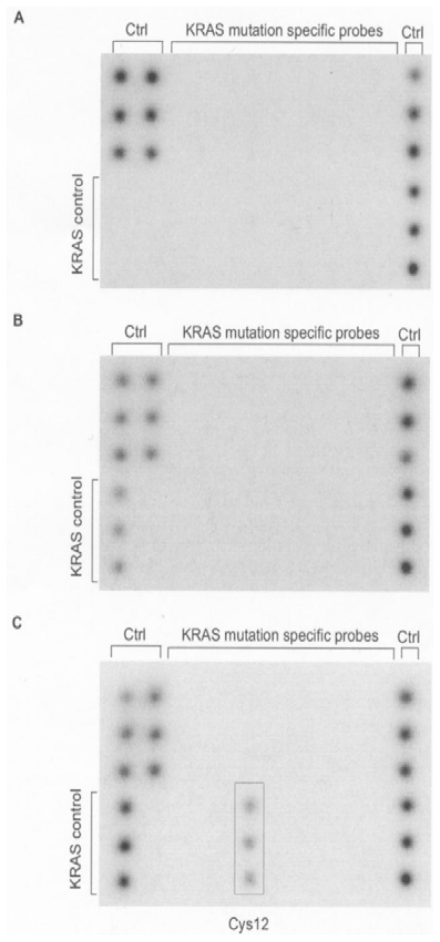
Chemiluminescent images after biochip hybridization are shown. Mutant-enriched PCR was performed using either 10 ng (**A**) or 100 ng (**B**) wild-type DNA (Colo320), and a mixture of 0.1 ng mutant (MIA Paca2) and 10 ng wild-type DNA (Colo320) (**C**). Control spots (Ctrl) were included to monitor for DNA extraction, suppression of *KRAS* wild-type amplification (KRAS control), and hybridization stringency.

The biochip assay’s limit for detecting *KRAS* mutations was exemplified using 0.1 ng of tumor cell line DNA mixed with 10 ng of wild-type DNA. Suppression of wild-type amplification by PNA clamping using 10 ng of wild-type template was found to be complete (Figure 1A), whereas *KRAS* mutation Cys12 contained in cell line MIA Paca2 was unambiguously identified, demonstrating an analytical sensitivity of 1% for the biochip assay (Figure 1C). Suppression of wild-type amplification using 100 ng of wild-type template was incomplete as indicated by the *KRAS* control spots (Figure 1B). The presence of *KRAS* wild-type PCR product, however, did not result in signals derived from *KRAS*-specific capture probes ensuring high specificity of the biochip assay.

Genomic DNA was isolated and amplified by mutant-enriched PCR from 81 primary NSCLC tumors including 48 adenocarcinomas, 30 squamous cell carcinomas and 3 large cell carcinomas. Biochip-based analysis of resulting PCR products identified 17 (21%) of 81 samples to carry a *KRAS* mutation of which 16/48 (33%) were adenocarcinomas and 1/30 (3%) was a squamous cell carcinoma (Table 1).

No mutation was detected in the 3 large cell carcinomas. Mutations were exclusively located in codon 12, with Asp12 (35%) being most frequent, followed by Cys12 (29%) and Val12 (18%) (Table 2). All mutations were confirmed by direct sequencing (data not shown).

With respect to disease stage, *KRAS* mutations were found in 33% (8/24) of patients with stage I, in 13% (2/15) of patients with stage IB, in 36% (4/11) of patients with stage IIA, and in 20% (3/15) of patients with stage IIIA (Table 1). No mutations were detected in patients with stages IIB and IIIB.

To determine the assay’s mutation detection limit in clinical specimens, mutant-enriched PCR was performed on genomic DNA isolated from 17 mutant NSCLC samples diluted 1:10 and 1:100 with wild-type DNA. Subsequent biochip hybridization was able to detect *KRAS* mutation present in all dilutions (data not shown), thereby supporting an analytical sensitivity of 1% for the biochip assay.

In this work, we analyzed 81 NSCLC tissue samples using a biochip assay designed for the sensitive detection of 10 mutations in codons 12 and 13 of the *KRAS* gene. Seventeen (21%) tumor samples contained a *KRAS* mutation, all of which were located in codon 12. This finding is in line with other studies that observed *KRAS* mutations among 20–33% of NSCLC patients with the majority of mutations being guanine to thymine transversions in codon 12 [12,13]. With respect to histotype, 16 (33%) of 48 adenocarcinomas and 1 (3%) of 30 squamous cell carcinomas were positive for a *KRAS* mutation. The increased prevalence of *KRAS* mutations in adenocarcinoma observed here corroborates earlier findings [12].

Using 10 ng of starting DNA, the biochip assay demonstrated a detection limit of 1% mutant sequence in a background of normal DNA. Therefore, this assay seems suitable for the detection of *KRAS* mutation in lung cancer because biopsies and FFPE sections are often small in size, thereby limiting template availability [13]. Similarly, low amount of cellular material is usually obtained by minimally invasive techniques such as transbronchial or transesophageal aspiration of the mediastinal lymph nodes [14].

More recent work used this biochip assay to screen for *KRAS* mutations in 85 DNA samples isolated from ovarian tissue [9]. In that study, all mutations detected by biochip hybridization were confirmed by sequencing after mutant-enriched PCR, thus being concordant with the results presented in this report. Subsequent studies including a total of 523 ovarian tissue samples indicate, that the biochip assay is fully compatible with *KRAS* mutation analysis in genomic DNA isolated from FFPE material [10,11]. This is of importance, because FFPE specimens are most commonly used for the detection of *KRAS* mutation.

Various molecular diagnostic methodologies such as DNA sequencing, capillary electrophoresis, amplification refractory mutation system (ARMS), and high resolution melting analysis (HRM) are available for *KRAS* mutation analysis [15–19]. All these methods have their advantages and disadvantages in terms of operational input, sample throughput, cost, and sensitivity.

Although labor-intensive and not very sensitive (*i.e*., analytical sensitivity of 20%), direct sequencing remains the gold standard for the detection of KRAS mutation in routine diagnostics [16,19]. A quantitative and more sensitive sequencing by synthesis approach (pyrosequencing) has been described demonstrating an analytical sensitivity of 5% on mixed DNA samples containing various amounts of mutant template [18]. In-tube real-time diagnostic procedures such as ARMS and HRM are rapid and sensitive to detect 1% and 5% to 6% mutant *KRAS* in a background of normal DNA, respectively [15,17], however, multiplexing possibilities are limited. Recently, a semiquantitative assay based on single nucleotide primer extension (SNaPshot) followed by capillary electrophoresis was shown to be a flexible alternative to direct sequencing for *KRAS* mutation analysis in colorectal FFPE DNA samples [16]. While being similar with respect to workflow, time to results, hands-on time, and costs, the SNaPshot assay is more sensitive, demonstrating a detection limit of 10% tumor cells. Moreover, SNaPshot offers a flexible assay design which might be easily modified to contain additional mutations.

Using 10 ng of starting DNA, the biochip assay described here allows simultaneous detection of 10 frequent *KRAS* mutations with a sensitivity of 1% mutant sequence in a background of wild-type DNA. The procedure is relatively fast (<6 h excluding DNA isolation), however, biochip hybridization is labor-consuming and data collection by chemiluminescence imaging lacks parallel processing ability, thereby limiting daily throughput to ≤24 samples. To evaluate the impact of DNA quality on template input and assay sensitivity, biochip analyses of DNA extracted from FFPE tissues are currently in progress.

## 3. Experimental Section

### 3.1. Tissue Samples and DNA Isolation

Primary NSCLC tumors (*n* = 81) were obtained from patients who had received surgical resections. There were 59 male and 22 female NSCLC patients, ages 42–82 years (mean, 64 years) at diagnosis. 24 patients had stage IA disease, 15 patients had stage IB disease, 11 patients had stage IIA disease, 14 patients had stage IIB disease, 15 patients had stage IIIA disease and 2 patients had stage IIIB disease. Histological subtypes of primary NSCLCs included 48 adenocarcinomas, 30 squamous cell carcinomas and 3 large cell carcinomas. Genomic DNA was isolated from frozen lung tumors by digestion with Proteinase K, followed by standard phenol-chloroform extraction and ethanol precipitation [20].

Patients gave their written informed consent and the study was approved by the local institutional review boards.

### 3.2. Mutant-Enriched PCR and Biochip Hybridization

Mutant-enriched duplex PCR and reverse-hybridization of PCR products to biochips was done as described earlier except for the fact that PCR was run for extra 10 cycles (*i.e.*, 45 cycles) [11]. Briefly, downstream primers were biotinylated and upstream primers were phosphorylated at the 5′-position. PCR was performed in a 25 μL reaction, containing 1× PCR Buffer (Qiagen, Hilden, Germany), 100 μM each deoxyribonucleoside triphosphate, 0.1 μM HLA-DRA primers, 0.25 μM KRAS primers, 2.84 μM PNA, 1 U Hot Star Taq Polymerase (Qiagen) and 10 ng DNA template. Amplifications were performed on a PE9700 cycler (Applied Biosystems, Foster City, CA) starting with an initial denaturation step at 95 °C for 15 min, then running for 45 cycles as follows: 94 °C for 1 min, 70 °C for 50 s, 58 °C for 50 s, 72 °C for 50 s, and a final extension at 72 °C for 7 min.

For biochip hybridization, 20 μL of PCR product was digested with 1 μL lambda exonuclease (New England BioLabs Inc., Ipswich, MA) at RT for 30 min. 10 μL of digested PCR product was then diluted in 200 μL of a solution containing 6× saline-sodium phosphate-EDTA (Sigma-Aldrich, St. Louis, MO) and 1 mL/L Tween 20 (Sigma) including a hybridization control target. Hybridization of the biochip was performed at 37 °C for 1 h in a conventional thermoshaker (Eppendorf AG, Hamburg, Germany). Without additional washing steps, the biochip was incubated for 15 min with streptavidin-peroxidase conjugate (Sigma) and thereafter rinsed with 1 mL 6× saline-sodium phosphate-EDTA (Sigma) containing 1 mL/L Tween 20 (Sigma). Upon addition of substrate (Chemiluminescent Peroxidase Substrate-3; Sigma), biochip signals were measured with a chemiluminescence detector developed for use with the biochip [9]. Images were displayed with the ImagQuant version 5.0 software (Molecular Dynamics), and genotyping calls were then made according to a set of quality criteria determined previously [21].

Genomic DNA isolated from tumor cell lines MIA Paca2 (Cys12) and Colo320 (wild-type) served as control templates and were included in each independent experimental set-up. 0.1 ng mutant genomic DNA mixed with 10 ng wild-type DNA and 10 or 100 ng wild-type DNA alone were used to monitor assay sensitivity and specificity, respectively. To determine the biochip assay’s limit for detecting *KRAS* mutation in NSCLC samples, PCR was performed on mutant DNA diluted 1:10 and 1:100 with wild-type DNA.

### 3.3. Dideoxy Sequencing

For DNA sequencing, *KRAS*-positive PCR products were column purified using the GenElute PCR CleanUp Kit (Sigma), and sequence analysis was performed on a ABI 310 automatic sequencer (Applied Biosystems) according to the manufacturer’s instructions (BigDye Terminator v1.1 Cycle Sequencing Kit; Applied Biosystems) using the *KRAS* sense primer.

## 4. Conclusions

Tailored therapy approaches have prompted the need for predictive biomarkers as drugs are costly and patients could be spared the side effects of pointless treatment. Recent data demonstrated that in NSCLC, the predictive role of *KRAS* is still inconclusive, and further studies should rely on methods optimized for the sensitive detection of *KRAS* mutation because biopsies and FFPE sections are often small in size, thereby limiting template availability. Using 10 ng of starting DNA, the biochip assay described here allows simultaneous detection of 10 frequent *KRAS* mutations with a sensitivity of 1% mutant sequence in a background of wild-type DNA, thereby making it a sensitive alternative to protocols currently in use for *KRAS* mutation testing on limited quantity samples.

## Figures and Tables

**Table 1:** Characteristics of 81 non-small cell lung cancer (NSCLC) specimens.

Characteristic		*n*	Mutated, *n* (%)
Total		81	17 (21)
Gender	Male	59	11 (19)
	Female	22	6 (27)
Pathology	Squamous cell carcinoma	30	1 (3)
	Adenocarcinoma	48	16 (33)
	Large cell carcinoma	3	0 (0)
Differentiation	Grade 1	3	0 (0)
	Grade 2	53	12 (23)
	Grade 3	20	3 (15)
	Unknown	5	2 (40)
Disease stage	IA	24	8 (33)
	IB	15	2 (13)
	IIA	11	4 (36)
	IIB	14	0 (0)
	IIIA	15	3 (20)
	IIIB	2	0 (0)
Pathologic tumor status	pT1	32	11 (34)
	pT2	40	5 (12)
	pT3	7	1 (14)
	pT4	2	0 (0)
Pathologic lymph node status	pN0	45	10 (22)
	pN1	22	5 (23)
	pN2	14	2 (14)

**Table 2:** Identity of 17 *KRAS* mutations detected by biochip hybridization.

Mutation	Amino acid	*n*	%
GGT→GAT	Gly12→Asp12	6	35
GGT→TGT	Gly12→Cys12	5	29
GGT→GTT	Gly12→Val12	3	18
GGT→GCT	Gly12→Ala12	2	12
GGT→AGT	Gly12→Ser12	1	6
GGT→CGT	Gly12→Arg12	0	0
GGT→ATT	Gly12→Ile12	0	0
GGT→CTT	Gly12→Leu12	0	0
GGC→GAC	Gly13→Asp13	0	0
GGC→TGC	Gly13→Cys13	0	0

	Total	17	100
